# Correction: A highly sensitive triazole-based perfectly water soluble novel bis-Schiff base reversible fluorescent-colorimetric chemosensor for fast detection of Pb^2+^ ions

**DOI:** 10.1039/d4ra90009j

**Published:** 2024-02-02

**Authors:** Vanshika Sharma, Sandhya Savita, Goutam Kumar Patra

**Affiliations:** a Department of Chemistry, Guru Ghasidas Vishwavidyalaya Bilaspur C.G. India patra29in@yahoo.co.in +91 7587312992

## Abstract

Correction for ‘A highly sensitive triazole-based perfectly water soluble novel bis-Schiff base reversible fluorescent-colorimetric chemosensor for fast detection of Pb^2+^ ions’ by Vanshika Sharma *et al.*, *RSC Adv.*, 2024, **14**, 3289–3303, https://doi.org/10.1039/D3RA06185J.

The authors regret that an incorrect version of Scheme 1 was included in the original article. The correct version of Scheme 1 is presented below.

**Scheme 1:**
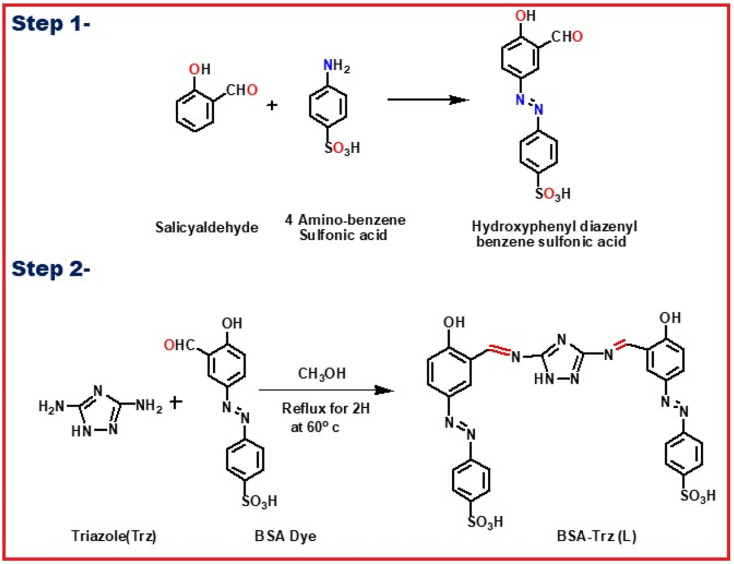
Synthetic procedure of the probe **L**.

The Royal Society of Chemistry apologises for these errors and any consequent inconvenience to authors and readers.

## Supplementary Material

